# Supplementary material for: Profiling Tight Junction Protein Expression in Brain Vascular Malformations
Source: Int J Mol Sci. 2025 May 9;26(10):4558. doi: 10.3390/ijms26104558 (PMC12111537; doi:10.3390/ijms26104558)
Supplement: Supplementary file 1 [file ijms-26-04558-s001.zip › ijms-3535738-supplementary.pdf]

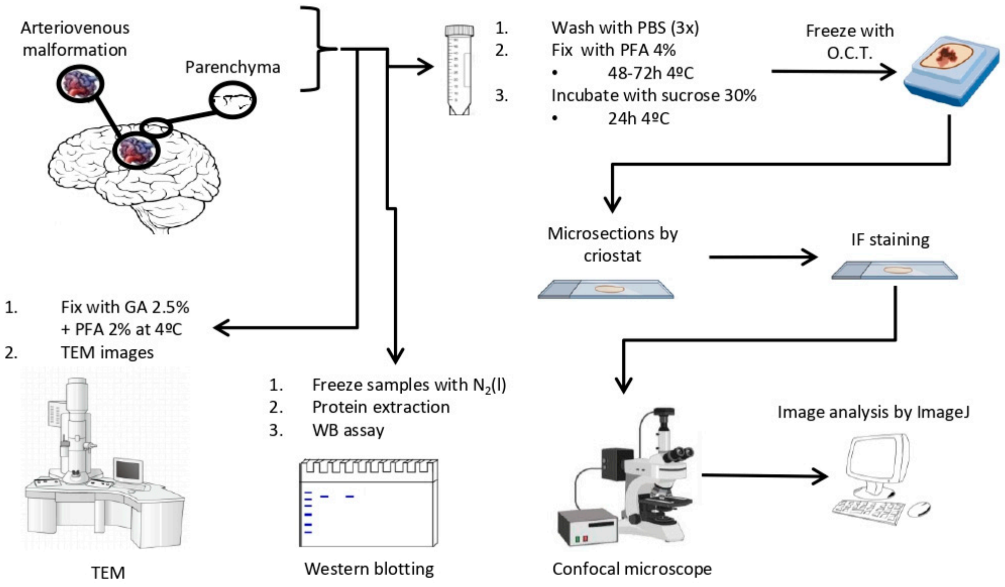

Supplementary Figure S1: Laboratory protocol desing.

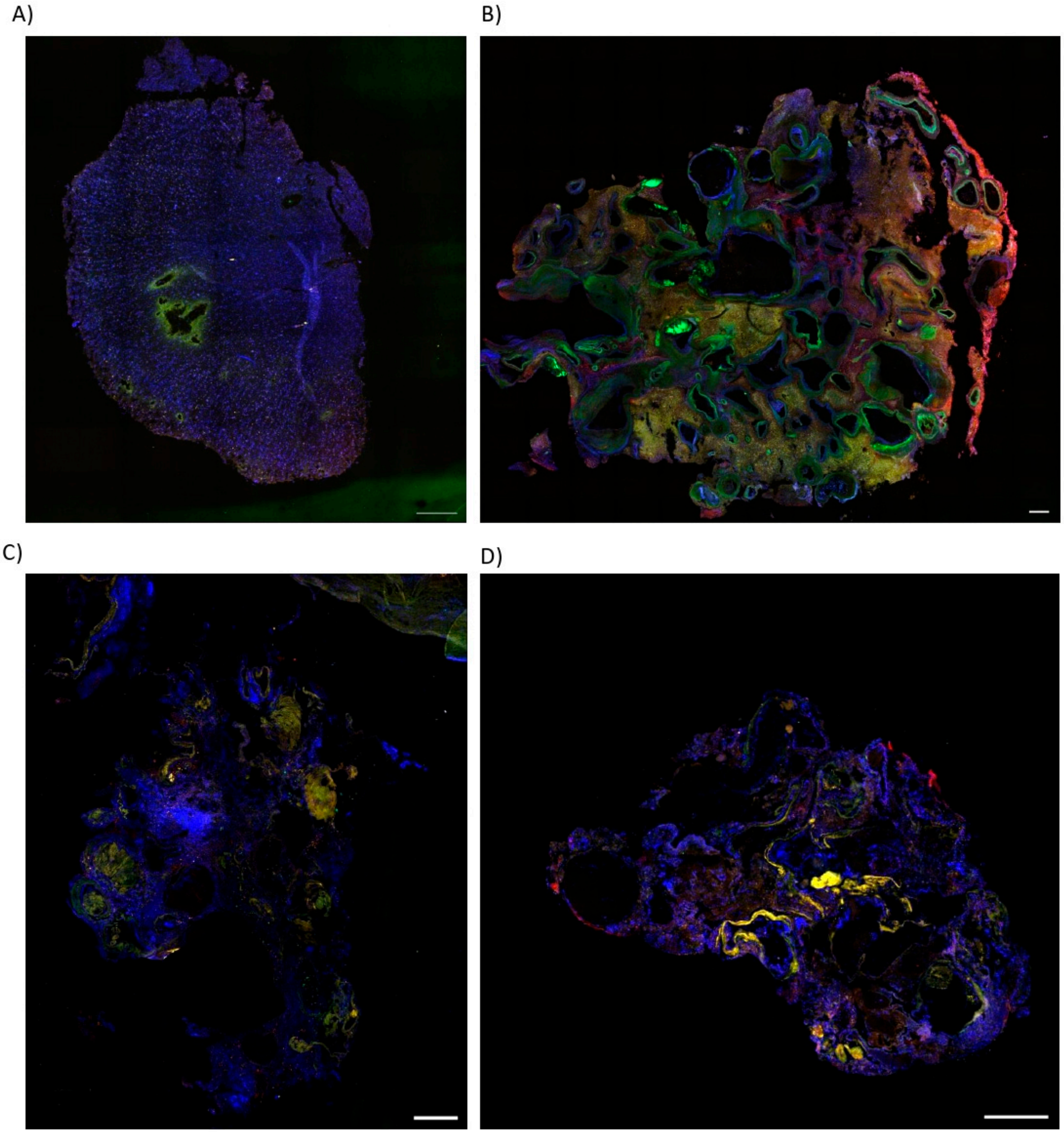

Supplementary Figure S2: TJ's protein expression analyzed by IF. Parenchymal (top, left) and bAVM (top, right) samples from bAVM non-ruptured, and CCM samples from ruptured (bottom, left) and non-ruptured (bottom, right). Claudin-5 (green), and Occludin(red) were used to mark tight junctions and endothelial cells, respectively. Nucleus was marked with Dapi (blue).

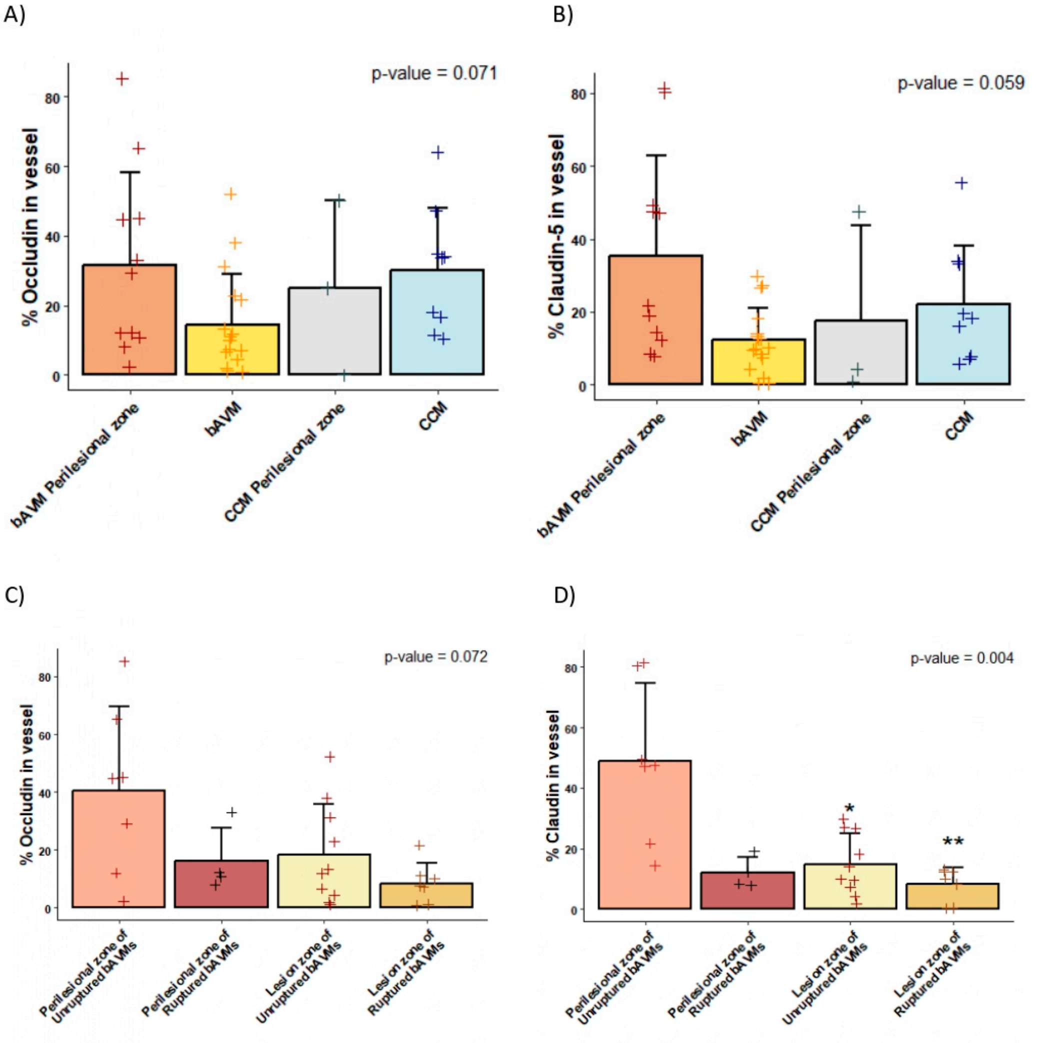

*Supplementary Figure S3: Quantification of TJ's protein expression analyzed by immunofluorescence. (A-B) Histograms of claudin-5 (right) and occludin (left) protein expression in vessels of the perilesional zone and the lesion specimens from bAVM and CCMs. (C-D) Histograms of claudin-5 (right) and occludin (left) protein expression in vessels of the perilesional zone and the lesion of bAVM specimens according to the rupture group. The expression was quantified by ImageJ. The p-value of ANOVA test is shown. bAVM: brain arteriovenous malformation; CCM: cerebral cavernous malformations. n=17 bAVMs; n=9 CCMs; n=11 bAMV perilesional zone; n=3 CCM perilesional zone. * p-value <0.05, **p-value<0.005.*

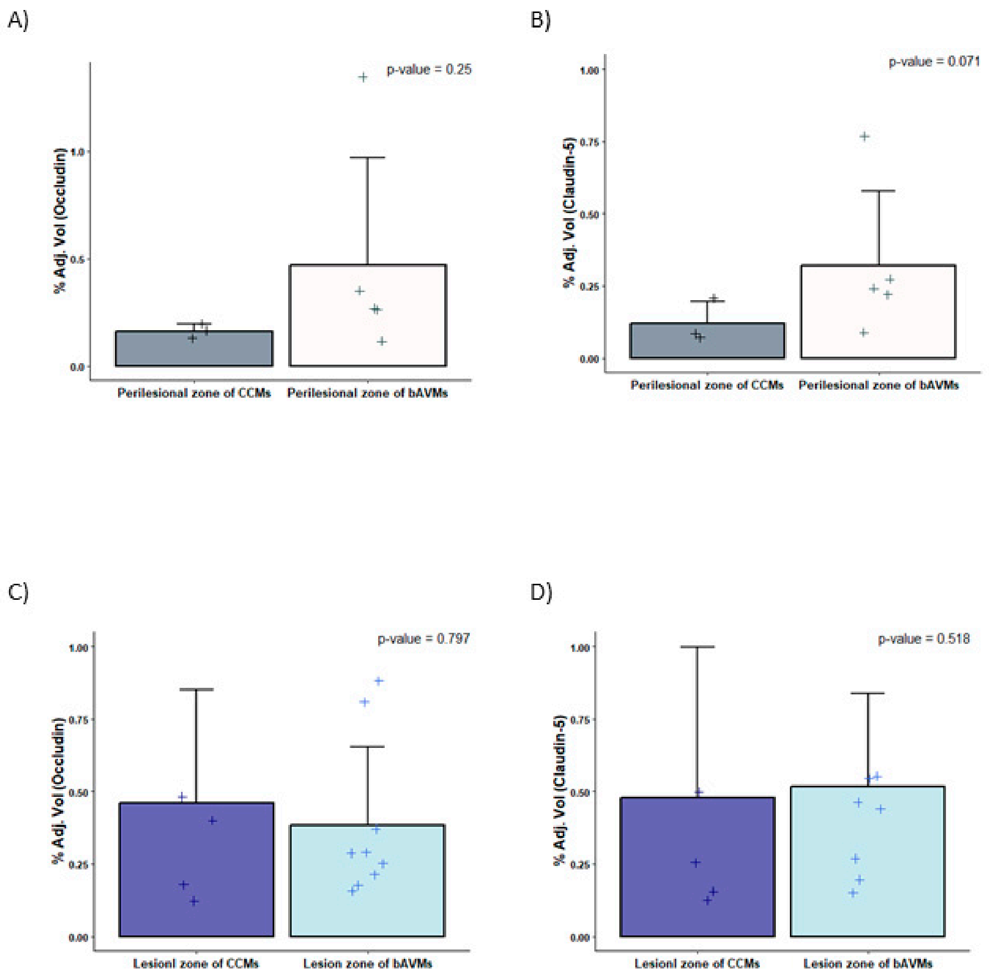

Supplementary Figure S4: Quantification of TJ's protein expression analyzed by Western Blotting. Histograms of claudin-5 (right panels) and occludin (left panels) protein expression in perilesional zone- (A-B) and malformation zone specimens (C-D), quantified by Onelab. bAVM: brain arteriovenous malformation; CCM: cerebral cavernous malformations;

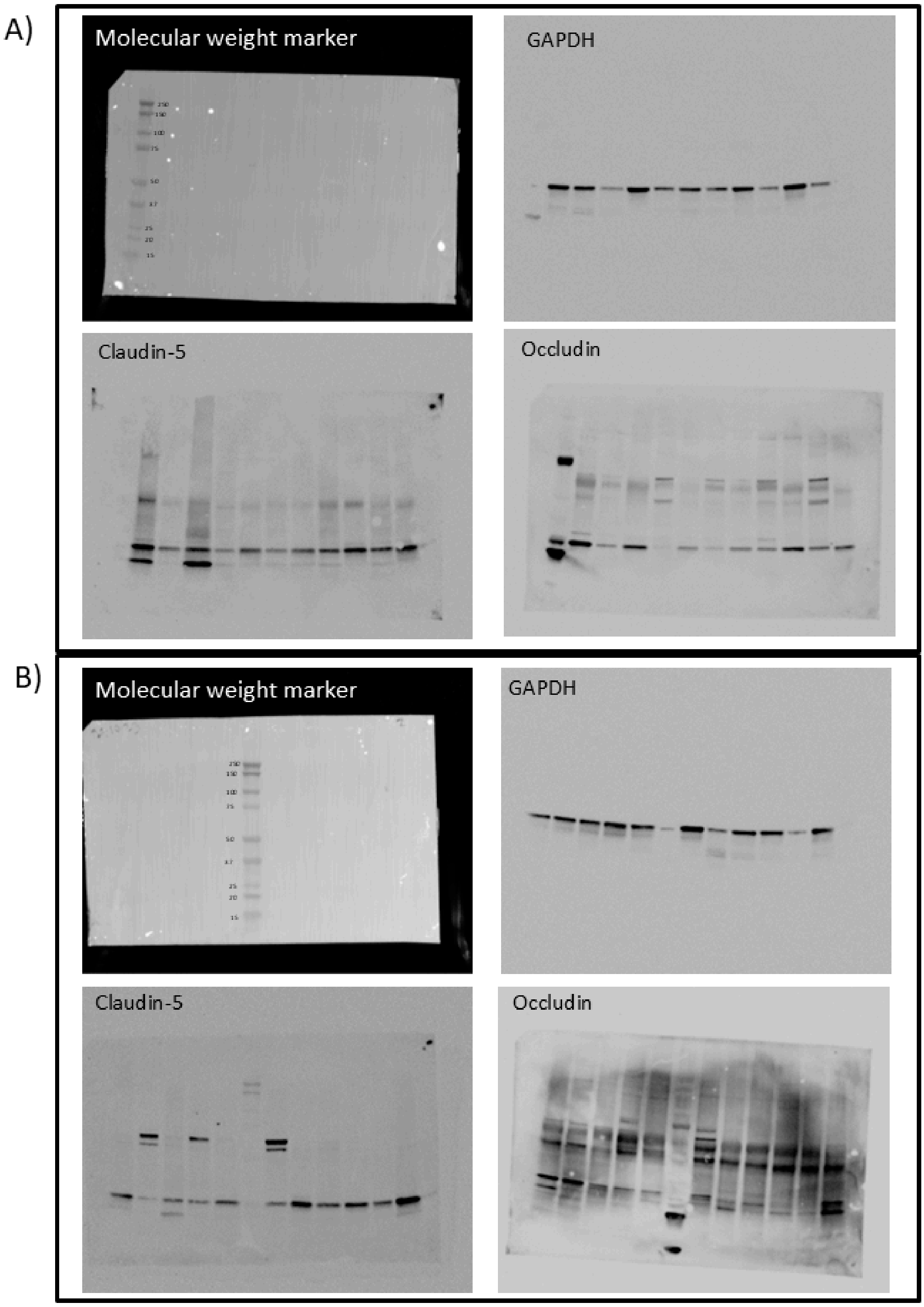

Supplementary Figure S5: Western blotting results. (A) Membrane 1. Order of simples: Marker, CCM2, Peri-CCM7, CCM7, Peri-bAVM7, bAVM7, Peri-bAVM8, bAVM8, Peri-bAVM17, bAVM17, Peri-bAVM33, bAVM33. (B) CCM3, Peri-CCM11, CCM11, Peri-CCM15, CCM15, Marker, Peri-bAVM13, bAVM13, bAVM3, bAVM4, bAVM5, bAVM10. bAVM: brain arteriovenous malformation; CCM: cerebral cavernous malformations; Peri: Perilesional zone.

Supplementary Table S1: Outcome of bAVMs patients. Data are given globally for all brain arteriovenous malformations (bAVM) and dichotomised according to the rupture status at diagnosis.

	All bAVM (n = 17)	Ruptured bAVM (n = 6)	Unruptured bAVM (n = 11)	p-value
Outcome (mRS) discharged, n (%)				0.512
0	6 (35,29%)	2 (33,33%)	4 (36,36%)	
1	6 (35,29%)	1 (16,67%)	5 (45,45%)	
2	4 (23,53%)	2 (33,33%)	2 (18,18%)	
3	1 (5,88%)	1 (16,67%)	0 (0,00%)	
4	0 (0,00%)	0 (0,00%)	0 (0,00%)	
5	0 (0,00%)	0 (0,00%)	0 (0,00%)	
6	0 (0,00%)	0 (0,00%)	0 (0,00%)	
Outcome (mRS) discharged, mean (SD)	1 (0.94)	1.33 (0.49)	0.818 (0.22)	0.292
Outcome (mRS) at 3 months, n (%)				1
0	5 (29,41%)	1 (16,67%)	4 (36,36%)	
1	4 (23,53%)	2 (33,33%)	2 (18,18%)	
2	6 (35,29%)	1 (16,67%)	5 (45,45%)	
3	2 (11,76%)	2 (33,33%)	0 (0,00%)	
4	0 (0,00%)	0 (0,00%)	0 (0,00%)	
5	0 (0,00%)	0 (0,00%)	0 (0,00%)	
6	0 (0,00%)	0 (0,00%)	0 (0,00%)	
Outcome (mRS) at 3 months, mean (SD)	1.29 (1.05)	1.66 (0.49)	1.09 (0.28)	0.292
Outcome (mRS) at 6 months, n (%)				1
0	5 (29,41%)	2 (33,33%)	3 (27,27%)	
1	2 (11,76%)	0 (0,00%)	2 (18,18%)	
2	4 (23,53%)	1 (16,67%)	3 (27,27%)	
3	0 (0,00%)	0 (0,00%)	0 (0,00%)	
4	0 (0,00%)	0 (0,00%)	0 (0,00%)	
5	0 (0,00%)	0 (0,00%)	0 (0,00%)	
6	0 (0,00%)	0 (0,00%)	0 (0,00%)	
Outcome (mRS) at 6 months, mean (SD)	0.91 (0.94)	0.67 (0.67)	1 (0.33)	0.630
Outcome (mRS) at 12 months, n (%)				1
0	4 (23,53%)	1 (16,67%)	3 (27,27%)	
1	2 (11,76%)	0 (0,00%)	2 (18,18%)	
2	4 (23,53%)	1 (16,67%)	3 (27,27%)	
3	0 (0,00%)	0 (0,00%)	0 (0,00%)	
4	0 (0,00%)	0 (0,00%)	0 (0,00%)	
5	0 (0,00%)	0 (0,00%)	0 (0,00%)	
6	0 (0,00%)	0 (0,00%)	0 (0,00%)	
Outcome (mRS) at 12 months, mean (SD)	1 (0.94)	1 (1)	1 (0.32)	1

Data are given as mean (standard deviation) unless otherwise specified.

Supplementary Table S2: Outcome of the control group (cavernomas, CCM).

	All CCM (n =16)	Ruptured CCM (n = 9)	Unruptured CCM (n = 7)	p-value
Outcome (mRS) discharged, n (%)				0.5556
0	4 (44,44%)	2 (28,57%)	2 (100,00%)	
1	1 (11,11%)	1 (14,29%)	0 (0,00%)	
2	2 (22,22%)	2 (28,57%)	0 (0,00%)	
3	2 (22,22%)	2 (28,57%)	0 (0,00%)	
4	0 (0,00%)	0 (0,00%)	0 (0,00%)	
5	0 (0,00%)	0 (0,00%)	0 (0,00%)	
6	0 (0,00%)	0 (0,00%)	0 (0,00%)	
Outcome (mRS) discharged, mean (SD)	1.22 (1.30)	1.57 (1.27)	0.00 (0.00)	**0.01709**
Outcome (mRS) at 3 months, n (%)				0.1429
0	2 (22,22%)	0 (0,00%)	2 (100,00%)	
1	2 (22,22%)	2 (28,57%)	0 (0,00%)	
2	2 (22,22%)	2 (28,57%)	2 (28,57%)	
3	1 (11,11%)	1 (14,29%)	1 (14,29%)	
4	0 (0,00%)	0 (0,00%)	0 (0,00%)	
5	1 (11,11%)	1 (14,29%)	1 (14,29%)	
6	0 (0,00%)	0 (0,00%)	0 (0,00%)	
Outcome (mRS) at 3 months, mean (SD)	1.41 (1.63)	1.86 (1.34)	0 (0)	
Outcome (mRS) at 6 months, n (%)				0.6789
0	3 (33,33%)	1 (14,29%)	1 (14,29%)	
1	3 (33,33%)	3 (42,86%)	3 (42,86%)	
2	1 (11,11%)	1 (14,29%)	1 (14,29%)	
3	0 (0,00%)	0 (0,00%)	0 (0,00%)	
4	1 (11,11%)	1 (14,29%)	1 (14,29%)	
5	0 (0,00%)	0 (0,00%)	0 (0,00%)	
6	0 (0,00%)	0 (0,00%)	0 (0,00%)	
Outcome (mRS) at 6 months, mean (SD)	1.22 (1.39)	1.57 (1.40)	0 (0)	
Outcome (mRS) at 12 months, n (%)				0.5714
0	2 (22,22%)	1 (14,29%)	1 (14,29%)	
1	3 (33,33%)	3 (42,86%)	3 (42,86%)	
2	1 (11,11%)	1 (14,29%)	1 (14,29%)	
3	0 (0,00%)	0 (0,00%)	0 (0,00%)	
4	1 (11,11%)	1 (14,29%)	1 (14,29%)	
5	0 (0,00%)	0 (0,00%)	0 (0,00%)	
6	0 (0,00%)	0 (0,00%)	0 (0,00%)	
Outcome (mRS) at 12 months, mean (SD)	1 (1.32)	1.29 (1.38)	0 (0)	
